# On Filling Teeth When Complicated with Inflammation of the Pulp

**Published:** 1853-10

**Authors:** C. Spence Bate

**Affiliations:** Plymouth, England.


### ARTICLE III.

### *On Filling Teeth when Complicated with Inflammation of the Pulp.* By C. SPENCE BATE, Esq., Plymouth, England.

THE numerous papers of interest which have appeared in the late numbers of the Journal of Dental Science under the head of "Filling over exposed lining membrane," &c., have induced me to send one or two cases which interested me much at the time. The *modus operandi*, I may also say, differs in some of its details from those given, and is, as far as I am aware, peculiar to myself.

The dentists in England unfortunately are exceedingly detached as individuals from each other. This, no doubt, originates from the want of some professional test, whereby we may distinguish the educated man from the less cultivated; but out of evil often proceeds good. Each member is thrown upon his own resources, in order to arrive at a successful treatment, and I have little doubt but that many in England, if they could be induced to publish their plans of treatment, would be able to exchange wrinkles of some value.

Jan'y 30.—Miss S., of London, came on a visit to her relations in Swansea, (where I resided and practiced for twelve years,) for her health, her constitution generally being debilitated. She called upon me relative to two central incisor teeth of the upper jaw, both of which were decayed, the right tooth loose, the gum inflamed and swollen, and an abscess developed beneath the lip. The whole had been for many days and still continued acutely painful. Upon my commencing to prepare the cavity for plugging, she expressed her doubt of its utility, because three times within two years had they been already done in the metropolis, but with no beneficial result. The filling soon became loose, and after a time came out, leaving the hole larger than before. She, therefore, would have preferred the entire removal of the tooth and an artificial substitute.

However, I proceeded with the operation, removed much of the decay but did not expose the pulp, though the decayed portion reached the central cavity of the tooth. I ordered a leech to the gum, an aperient and rest, and dismissed her after having filled the tooth with *gutta percha stopping*.

What I call *gutta percha stopping* is a mixture I make myself, of plaster of paris and gutta percha, mixed before the fire until the latter will take no more without crumbling. This I find a useful temporary stopping, and after it has been in the mouth a few minutes presents no excessive contrast with the tooth.

February 10.—On this day my patient again visited me. I found the tooth firmer, with less swelling. The leech had been misapplied and took only on the lip; the tumor got worse, and a second leech was more successful, removing the pain and bringing down the swelling. I opened the abscess on the gum, filled permanently the left incisor, which was not complicated in the inflammatory action which had attacked the right.

March 5.—Found the tooth firm and free from pain, abscess gone, a slight redness with scarcely perceptible swelling remained in its place, but which my patient informed me had not been so much the day before as now. The temporary stopping was not well in. She had suffered no pain since I last saw her, but the tooth had lost its color, looking a little gray, the pulp was dead and some dark tissue remained, which was removed by boring into the pulp cavity, then plugged the tooth, adopting a plan recommended in one of the American journals, of putting in a piece of white paper to prevent the gold from being seen through the transparent enamel. I separated the two incisors with a file, and ordered a leech to the spot where the abscess had previously been, more as a precaution than from any immediate necessity.

September 16.—Filled to-day some teeth for the above patient's uncle, to whom she had been on a visit while in Swansea. He informed me that she had been perfectly comfortable since I saw her last, and had now returned home.

Miss. J. upon hearing that I was about to leave Swansea asked me to examine her mouth. Among other things which I thought necessary to be done, I found a tooth which I had plugged many years before, the gold having shown signs of wearing from mastication, I therefore removed it. I found the cavity healthy, except a little softening of dentine near the surface where a fragment of enamel had broken away. Sometime since, she had been conscious of some uneasiness about the tooth. Removed the incipient caries and manipulated a little, chiefly with a view of improving the shape of the cavity, in order to give durability to the plug. While using a fine broach for the purpose and pressing with some force, it slipped from its place and broke through the thin wall of dentine into the pulp cavity. The immediate result of such clumsiness on my part was pain of rather a severe character, but which fortunately was not continuous; a small quantity of blood oozed through the perforation. Over this I placed a thin solution of gutta percha dissolved in chloroform, and then pressed in the gold and completed the stopping.

The next morning, to my great discomfort, the lady reported that she had suffered much pain and could not drink cold water, and warm tea also gave her pain. I begged for two days trial, as pain often continues for a short time after the operation, the result of irritation from cutting dentine in a healthy state.

At the termination of the period agreed upon, she stated that the pain was less acute, but still more than she could possibly bear. Consequently, I removed the plug, reduced a gnawing pain by the topical application of chloroform, and dismissed her with a little cotton in the tooth.

March 10, a week after. The tooth was so far restored that she feared to have it filled again. But the tooth, though easy, I found, upon examination after the removal of the plug, to be exquisitely sensitive to the slightest touch of the instrument. In order to defend the living dentine from the rude contact of the gold, I cut a thin section of dentine from the tooth of a walrus, and to render it pliant, I dipped it for a few minutes in hydrochloric acid, after washing it well to free it from all trace of acid, I cut it to the proper form, laid it at the bottom of the cavity and pressed the gold upon it. The commencement of the operation was painful, but was ultimately successful, so that when tested with cold water, it remained free from pain.

On the 24th, that is thirteen days after, my patient called and reported that for some days the tooth was easy, but suddenly (to use her expression) "something in the tooth seemed to give way," since which it had been very painful, but the night before the ache appeared to settle in the second under molar, which had been filled long previously, but which upon being carefully examined, was found to have a small carious spot which burrowed into the tooth and became complicated with the cavity already plugged. The latter I tore out and refilled the tooth, which bore the operation well, though severe pain was caused at first by the application of tra. camph. to the denuded cavity; but no inconvenience was felt upon pressing in the gold—which was leaf. Presuming that the pain felt was caused by this last tooth, I sent my patient away without meddling with the upper molar, the tooth in question.

On the 26th, I found the last mentioned tooth (upper molar) still severely annoying her. I again removed the filling, upon which, we were both cognizant of a slight but offensive effluvia. The gold which came in contact with the ivory-plate was slightly darkened in color, which convinced me that, however careful I might have been in washing the plate, still some acid must have remained to the disadvantage of the tooth and risk of the operation, although the ivory plate was so perfectly and firmly attached to the bottom of the cavity, that I could not immediately remove it.

I now refilled the tooth temporarily with gutta percha stopping, which remained in without any deterioration until the 9th of September, when I found the small communication, through the floor of the cavity closed up by dentine. I now filled the tooth without any false bottom to the cavity. It bore the operation with impunity, and remained well when I last heard of it, and I have no doubt, but that it will continue to be permanently successful.

Mr. M. aged 16, had a first under molar, decayed upon the masticating surface, which had been unskillfully filled some weeks before, and from the period of the operation to the day he consulted me, he had no entire cessation from pain. I removed the plugging and cut out the caries, much of which remained round the walls of the cavity, carefully made and adapted to the bottom a thin section of ivory,* sufficiently stout to bear the pressure of the operation, from a tooth of the hippopotamus, and then packed in the filling. The tooth was immediately free from pain, and continued so up to the time of my leaving Swansea, which was many months after the operation.

I could give the history of many such cases as this last, viz. Painful teeth stopped with an ivory cap or false bottom to the cavity. I have for a long time preferred ivory caps over the pulp to gold, as being more congenial to the nature of a tooth, being itself a non-conductor of heat and cold, and therefore capable of being brought into close proximity with the nerve itself, an organ which, in my opinion, should seldom be destroyed in young persons.

In individuals past maturity, the pulp is a mere remnant of an organ and is itself often deprived of vitality, being strangu-

*This ivory was not treated with acid.

lated by the narrowing of the orifice at the extremity of the fang; but in young persons it is full of life and activity, and capable of restoration. When partially diseased, moreover, its passage through the extremity of the fang is so large, that, to destroy the pulp is to destroy, at all events to risk, the attaching membranes of the tooth, for it is difficult to limit the extent of inflammation we wish to excite so as to define the point where slough of the pulp shall take place, and to fail, is to excite a morbid action in the lining membranes contiguous with the external surface of the tooth, to destroy which, is to make the tooth an extraneous body. The sooner then that it is removed, the better for the general health.

The plan which I have generally adopted, in order to relieve the pain consequent upon an inflamed pulp, is first to observe, if the tooth be tender on being brought into contact with those in the opposite jaw; if this be the case, without any distinct looseness in the tooth itself, the result of an inflammatory action in the alveolar membranes. The tooth should be isolated from its neighbor, by the use of a file which should distinctly preclude any contact so as to interfere with the natural articulation of the tooth itself when the two jaws meet. In such cases, the use of the file to an exceedingly painful tooth, is less disagreeable than the same application to a more healthy one, and relief is apparent immediately upon the decided separation of the tooth from the other. Upon this being completed, I return to the cavity and proceed to remove as much as possible of the superincumbent gangrenous osseous tissue, by taking off the pressure from the confined and swollen pulp, to allow it to have free scope for its increased dimensions. This is easily done by means of sharp excavators; but should the tissue be hard, the pulp being exposed only by an almost impervious opening, I generally use a rose drill hollowed in the centre, or a broach similarly constructed so that I can cut all round the chamber without injuring the pulp. Having proceeded sufficiently deep, the bottom of the cavity should, if possible, be removed in one piece, care being taken that no splinter or spiculæ of dentine be left in contact with the irritated pulp; for I believe this is not unfrequently the cause of an unsuccessful treatment.

Having giving room to the pulp to relieve itself by swelling, I endeavor to reduce any irritation which may continue by the application of either saturated tra-camphoræ, chloroform or camphorated chloroform, to both the exposed pulp and soft tissues of the mouth near the seat of pain.

Having obtained relief, which I think is generally certain of being procured under careful and light manipulation in no lengthened period; I warm a little gutta percha stopping, over a spirit lamp and press it gently in, being cautious not to press upon the already too excited pulp, and secondly, careful to be certain that it hermetically seals up the cavity, and precludes the admission of external irritants. Should there be any amount of inflammation about the gums, special cases may require the local application of a leech. The use of a lancet, except for the exit of confined pus, and some very extreme cases, I do not admire, believing that the small quantity of blood so drawn off will seldom compensate for the irritation produced, together with the more rapid and increased tendency of blood to the part, in order to supply the place of that which has been taken away.

A week after, unless pain brings them earlier, I generally expect to see the tooth under treatment again, and judge according to circumstances, how far it may be advisable to fill the tooth immediately or postpone the operation.

Many have attempted to express which they think the most successful way of manipulating with gold, but as far as my own experience has gone, I feel myself unable to tell, until I see the cavity I have to fill, how to handle the material. One of the most favorite modes with me is, in large cavities, to put in the gold either in the form of a bundle of rods, the foil being rolled into the form of wire or doubled up into flat plates. The latter I prefer where the walls of the cavity are not too strong, since they can be laid side by side, each pressed laterally, without any excessive force. I think this latter has been one of my most successful methods, where there has been some anticipated difficulty, such as a shallow or anomalously formed cavity. *Apropos* to a shallow cavity, I would remark, that a hollow rose-drill will be found valuable, since it will excavate the tissue deeper around the circumference of the cavity, and permit the dentine to remain in the centre where it is most needed, the angle formed by this means at the circumference at the bottom of the cavity, will assist rather than otherwise to add firmness to the plug.

Much has been said as to the relative value of the different materials used in stopping. Gold foil is too well known to require a remark since it stands, by experience, pre-eminent.

A word on spongy gold, which Mr. Tomes, when he first advocated it, thought would rapidly supersede the use of foil. Of this material, there are three sorts which have been supplied to me. The first was in the form of a coarse granular powder. It was made by Makins, and supplied me by Messrs. Ashton. With this I filled several teeth, some of which I never heard of again—but two I have lately seen, being a year after they were done. I was very glad to have the opportunity of removing the spongy gold and replacing the plug with foil.

The second form is of Messrs. Ash's own preparation. It is in the form of small nodules of brown porous-looking gold. This, for some time, I was rather inclined to like, I filled with it many teeth, but ultimately came to the conclusion that it was uncertain. Two conditions were absolutely required, a *large cavity* and *perfect freedom from all moisture*, although I could point to fillings of this kind which for firmness and strength, I could not surpass with any but the finest foil, yet the uncertainty of the operation induced me to abandon it also. The third is in the form of stout leaf, of this, I received but a small quantity through the kindness of my friend and relative, Mr. Rogers, of Newbury. This he had direct from Mr. Makin, who, now, I believe, does not continue to supply it from press of other business. With this I filled only two teeth, one of which I have been able to keep in sight. Up to the present time, this filling is as perfect as when put in, but I think I could have made a better finished plug with foil. This might and probably does arise from want of experience in its manipulations; but the great drawback appears to be in the fact, that, wherever the instrument has pressed, no cohesion of particles can take place, thus precluding necessary manipulation. This, together with deterioration to the plug, by the accidental presence of saliva, particularly, if much mucus be present, is more than compensation, I fear, for the solitary advantage proposed, the greater ease with which it is inserted.

The next stopping which is used, claims attention, if not from its quality, at least from the *quantity* that is used. I mean the mercurial pastes, which no doubt in England are too greatly abused; an abuse that will exist until mammon cease to reign. But I cannot say, that I think it can altogether be superseded, and to substantiate my opinion, I will mention the two following cases:

Twelve years since, Miss A. came to me at Swansea, suffering under the most excruciating agony in the first under molar. Relief was given by the excavation of the gangrenous dentine, and the topical application of camphorated spirits. Without doing more, I put into the cavity, which embraced the whole of the masticating surface, a ball of silver amalgam. A week after it came on, I took it out, the tooth being totally free from pain since the first manipulation, I continued the operation of excavating the decay, and replaced a plug by a similar material. This continued in firm, strong, and *ugly*, until last year the tooth was extracted by a surgeon by mistake for a posterior neighbor.

The second case is more recent, and one which, by all the rules of our science, should be filled with gold. It was a front upper lateral, decayed at the back quite through to the enamel, and when cleared out for filling was almost severed in too, the quantity of dentine which remained to unite the apex of the crown with the base being only a small portion on one side. Since the filling could be seen through the transparent enamel, I fitted very accurately at the bottom of the cavity a thin section of ivory, which I afterward changed for white paper, and then put in the gold in the form of thin flat plates, pressing them laterally so that they should all stand vertical to the cavity. I succeeded in getting the gold firmly in, and was proceeding to consolidate the mass when having spent about an hour operating as cautiously as I thought the case demanded, I saw a crack fly across the enamel. What was I to do? Others might have succeeded better; but what I did is soon told. I took out the gold, an operation itself which greatly risked the tooth, placed a piece of paper and filled with ash-cement, (which I apprehend to be very analogous to that given by Mr. Robertson, in the Journal for July.) Some months have passed away, and the tooth, I have heard, has been so far preserved, both with color and comfort.

It appears to me that owing to the extremely fragile nature of the remaining structure in both these cases, if cement had not been used, two teeth would have been sacrificed. But as I before remarked, all this is no excuse for the great lengths to which the abuse of amalgams have been carried. Had there been no amalgam, the empirical dentists would not have been so numerous, and if upon no other ground than for the elevation of a professional standard, I would urge the expulsion of mercurial fillings from the practice of dental surgery.

The last stopping which is much used is tin, this I hold in low estimation, since it has not one redeeming point but cheapness. If it is used on a masticating surface, it will wear rapidly away, if upon a lateral, it becomes dark, often nearly black, if it be used for a large cavity, it, for a long time, by the action of the saliva, gives an unpleasant liquorish taste to the mouth.

My own feeling, is to expunge it entirely from adult practice. I think, perhaps, sometimes it might be made available for the preservation of the teeth of the deciduous set, since they are only valuable for a time, and it would generally be well to avoid placing in the mouth of young children any mercurial compound, but even here I often use gutta percha stopping in preference to others.

8 *Mulgrave Place, Plymouth, England,*
*October* 23*d*, 1852.

